# Supplementary material for: Relative Excess Risk of Metabolic Syndrome Due to Interaction Between Handgrip Strength and Dietary Patterns Among Korean Youth
Source: Nutrients. 2025 Jul 10;17(14):2282. doi: 10.3390/nu17142282 (PMC12300056; doi:10.3390/nu17142282)
Supplement: Supplementary file 1 [file nutrients-17-02282-s001.zip › nutrients-3727886-supplementary.pdf]

Supplementary materials of,

Relative Excess Risk of Metabolic Syndrome Due to Interaction Between Handgrip Strength and Dietary Patterns Among Korean Youth

Seong Woong Yoon[1], Hunju Lee[2], Hyowon Choi[2*], Yunkoo Kang[3*]

1 Department of Medicine, Yonsei University Wonju College of Medicine, Wonju, Korea; yoonsw0429@naver.com
2 Department of Prevention Medicine, Yonsei University Wonju College of Medicine, Wonju, Korea; H.L. hjlee5371@gmail.com; H.C., wowo0226@gmail.com
3 Department of Prevention Medicine, Yonsei University Wonju College of Medicine, Wonju, Korea; monkeydluffy@yonsei.ac.kr

*Correspondence: wowo0226@gmail.com (H.C.), monkeydluffy@yonsei.ac.kr (Y.K.);

Supplementary material Table S1. Multivariable logistic regression stratified by sex, Unweighted

		Male	Female
**Metabolic Syndrome**			
Handgrip Strength	Normal	1 (Ref)	1 (Ref)
	Low HGS	1.135 (1.097~1.174)	1.117 (1.089~1.147)
Food style	Balanced	1 (Ref)	1 (Ref)
	Processed Fat	1.027 (1.000~1.053)	0.992 (0.971~1.012)
	Western	1.017 (0.991~1.044)	0.988 (0.968~1.009)
**Central obesity**			
Handgrip Strength	Normal	1 (Ref)	1 (Ref)
	Low HGS	1.557 (1.471~1.647)	1.333 (1.264~1.406)
Food style	Balanced	1 (Ref)	1 (Ref)
	Processed Fat	1.021 (0.975~1.069)	1.001 (0.959~1.044)
	Western	1.032 (0.985~1.082)	1.022 (0.979~1.066)
**Hypertension**			
Handgrip Strength	Normal	1 (Ref)	1 (Ref)
	Low HGS	1.122 (1.069~1.177)	1.021 (0.991~1.052)
Food style	Balanced	1 (Ref)	1 (Ref)
	Processed Fat	1.016 (0.980~1.053)	1.001 (0.978~1.024)
	Western	1.034 (0.997~1.072)	1.009 (0.986~1.032)
**Hyperglycemia**			
Handgrip Strength	Normal	1 (Ref)	1 (Ref)
	Low HGS	1.055 (0.985~1.130)	1.137 (1.078~1.200)
Food style	Balanced	1 (Ref)	1 (Ref)
	Processed Fat	1.046 (0.995~1.101)	1.000 (0.959~1.041)
	Western	1.036 (0.984~1.090)	1.006 (0.965~1.048)
**Low HDL-C**			
Handgrip Strength	Normal	1 (Ref)	1 (Ref)
	Low HGS	1.211 (1.133~1.293)	1.181 (1.095~1.274)
Food style	Balanced	1 (Ref)	1 (Ref)
	Processed Fat	0.991 (0.943~1.041)	1.023 (0.965~1.083)
	Western	1.003 (0.954~1.054)	1.002 (0.946~1.061)
**High TG**			
Handgrip Strength	Normal	1 (Ref)	1 (Ref)
	Low HGS	1.118 (1.056~1.184)	1.087 (1.029~1.149)
Food style	Balanced	1 (Ref)	1 (Ref)
	Processed Fat	0.998 (0.957~1.042)	1.010 (0.969~1.053)
	Western	1.006 (0.964~1.050)	0.982 (0.942~1.023)

HGS, handgrip strength; HDL-C, high-density lipoprotein cholesterol; TG, triglyceride.

Supplementary material Table S2. Interaction effects of handgrip strength and dietary patterns on metabolic syndrome and its components, unweighted

		Male		Female	
**Metabolic Syndrome**		OR	RERI	OR	RERI
Handgrip strength	Food Style				
Normal	Balanced	1(Ref)		1(Ref)	
Normal	Processed Fat	1.007 (0.981~1.033)		0.991 (0.970~1.012)	
Normal	Western	1.005 (0.979~1.032)		0.984 (0.964~1.005)	
Low	Balanced	1.016 (0.957~1.078)		1.079 (1.029~1.132)	
Low	Processed Fat	1.275 (1.201~1.353)	-0.27 (-0.33~-0.21)	1.131 (1.076~1.189)	-0.14 (-0.19~-0.09)
Low	Western	1.142 (1.079~1.208)	-0.14 (-0.19~-0.08)	1.112 (1.066~1.160)	-0.13 (-0.17~-0.09)
**Central obesity**					
Normal	Balanced	1(Ref)		1(Ref)	
Normal	Processed Fat	1.000 (0.957~1.045)		1.022 (0.979~1.066)	
Normal	Western	1.010 (0.966~1.056)		1.027 (0.984~1.072)	
Low	Balanced	1.328 (1.203~1.467)		1.377 (1.251~1.516)	
Low	Processed Fat	1.753 (1.587~1.937)	-0.75 (-0.85~-0.65)	1.279 (1.156~1.415)	-0.26 (-0.36~-0.16)
Low	Western	1.629 (1.482~1.791)	-0.62 (-0.71~-0.52)	1.398 (1.284~1.523)	-0.37 (-0.45~-0.29)
**Hypertension**					
Normal	Balanced	1(Ref)		1(Ref)	
Normal	Processed Fat	1.026 (0.989~1.065)		1.009 (0.985~1.033)	
Normal	Western	1.035 (0.996~1.074)		1.012 (0.988~1.037)	
Low	Balanced	1.159 (1.065~1.261)		1.054 (0.998~1.113)	
Low	Processed Fat	1.085 (0.996~1.181)	-0.06 (-0.14~0.03)	0.987 (0.932~1.045)	0.02 (-0.03~0.08)
Low	Western	1.189 (1.096~1.289)	-0.15 (-0.23~-0.07)	1.039 (0.990~1.090)	-0.03 (-0.07~0.02)
**Hyperglycemia**					
Normal	Balanced	1(Ref)		1(Ref)	
Normal	Processed Fat	1.024 (0.971~1.080)		1.022 (0.979~1.066)	
Normal	Western	1.024 (0.971~1.		1.008 (0.966~1.	

		081)		052)	
Low	Balanced	0.945 (0.837~1.067)		1.201 (1.091~1.322)	
Low	Processed Fat	1.216 (1.078~1.373)	-0.19 (-0.31~-0.07)	1.016 (0.918~1.124)	0.01 (-0.09~0.11)
Low	Western	1.074 (0.957~1.205)	-0.05 (-0.16~0.07)	1.209 (1.110~1.317)	-0.20 (-0.29~-0.12)
**Low HDL-C**					
Normal	Balanced	1(Ref)		1(Ref)	
Normal	Processed Fat	0.985 (0.936~1.037)		1.026 (0.965~1.089)	
Normal	Western	0.998 (0.948~1.052)		0.997 (0.938~1.060)	
Low	Balanced	1.159 (1.031~1.302)		1.144 (0.999~1.312)	
Low	Processed Fat	1.250 (1.112~1.406)	-0.27 (-0.38~-0.15)	1.218 (1.055~1.406)	-0.19 (-0.33~-0.05)
Low	Western	1.203 (1.076~1.345)	-0.20 (-0.32~-0.09)	1.209 (1.071~1.365)	-0.21 (-0.33~-0.09)
**High TG**					
Normal	Balanced	1(Ref)		1(Ref)	
Normal	Processed Fat	0.980 (0.937~1.024)		1.012 (0.968~1.057)	
Normal	Western	0.993 (0.949~1.039)		0.981 (0.939~1.025)	
Low	Balanced	1.000 (0.904~1.107)		1.077 (0.976~1.189)	
Low	Processed Fat	1.209 (1.093~1.338)	-0.23 (-0.33~-0.13)	1.110 (1.001~1.232)	-0.10 (-0.20~0.00)
Low	Western	1.121 (1.018~1.235)	-0.13 (-0.22~-0.03)	1.072 (0.982~1.170)	-0.09 (-0.18~-0.01)

OR, odds ratio; RERI, relative excess risk due to interaction; HDL-C, high-density lipoprotein cholesterol; TG, triglyceride.
